# Vancomycin-based tracers guiding in situ visualization of bacteria on osteosynthesis devices and surgical debridement

**DOI:** 10.1007/s00259-025-07249-4

**Published:** 2025-04-02

**Authors:** Gerbren B. Spoelstra, Philip H. Elsinga, Jan Maarten van Dijl, Johannes H. van Snick, Ben L. Feringa, Andor W. J. M. Glaudemans, Bas Keizers, Schelto Kruijff, Wiktor Szymanski, Marleen van Oosten, Frank F. A. IJpma

**Affiliations:** 1 Department of Nuclear Medicine and Molecular Imaging, University of Groningen, University Medical Center Groningen, Hanzeplein 1, Groningen, 9713 GZ The Netherlands; 2 Department of Medical Microbiology and Infection Prevention, University of Groningen, University Medical Center Groningen, Hanzeplein 1, Groningen, 9713 GZ The Netherlands; 3 Stratingh Institute for Chemistry, University of Groningen, Nijenborgh 4, Groningen, 9747 AG The Netherlands; 4 Department of Surgery, University of Groningen, University Medical Center Groningen, Hanzeplein 1, Groningen, 9713 GZ The Netherlands; 5 Department of Molecular Medicine and Surgery, Karolinska Institutet, Stockholm, Sweden; 6 Department of Radiology, University of Groningen, University Medical Center Groningen, Hanzeplein 1, Groningen, 9713 GZ The Netherlands; 7 Department of Medicinal Chemistry, Photopharmacology and Imaging, University of Groningen, Groningen Research Institute of Pharmacy, Antonius Deusinglaan 1, Groningen, 9713 AV The Netherlands; 8 Department of Trauma Surgery, University of Groningen, University Medical Center Groningen, Hanzeplein 1, Groningen, 9713 GZ The Netherlands

**Keywords:** Bacterial imaging agents, Positron emission tomography, Near infrared fluorescence, Fluorescence guided surgery, Bacterial biofilm, Fracture-related infections, Vancomycin, Debridement, Human post-mortem

## Abstract

**Purpose:**

Bacterial infections associated with musculoskeletal injuries are challenging to detect and distinguish from sterile inflammation. Here we present the combined first-time application of a bacteria-targeted positron emission tomography (PET) tracer and a near-infrared fluorescent tracer to detect infected osteosynthesis implants and guide surgical treatment.

**Methods:**

To this end, osteosynthesis plates covered with bacterial biofilm and pre-incubated with [^18^F]PQ-VE1-vancomycin for PET imaging and/or vancomycin-IRDye800CW for optical imaging were fixed to post-mortem human tibiae and femora. PET/CT and fluorescence imaging were used to quantify the bacterial load before and after surgical debridement.

**Results:**

Pre-debridement, PET imaging showed a significant 2.2-fold higher tracer uptake on biofilm-covered plates compared to plates without biofilm (*p* < 0.001). Post-debridement, the PET signal was marginal, demonstrating effective biofilm removal. Fluorescence-guided surgery enabled real-time visualization and removal of bacterial biofilms.

**Conclusion:**

Combined preoperative PET and intraoperative fluorescence imaging with vancomycin-based tracers allows noninvasive detection and real-time infection management, as demonstrated by these preliminary findings.

## Introduction

Fracture-related infection (FRI) is a severe complication after musculoskeletal injury with substantial personal and societal impact [1–3]. A major clinical challenge related to FRI is the formation of bacterial biofilms on implanted osteosynthesis devices. Biofilm-embedded bacteria present high resistance to both the human immune defenses and antimicrobial therapy. Early detection and accurate diagnosis of FRI are, therefore, of prime importance for optimal patient outcome. However, the diagnosis of FRI is complicated by the fact that bacterial infections are difficult to distinguish from sterile inflammation caused by factors such as soft tissue injury, fractured bone, surgery and implanted osteosynthesis devices. Moreover, during surgical revision for suspected FRI, it is difficult to distinguish infected tissue from healthy tissue. As a consequence, surgical implant debridement may be inadequate and patients may have to undergo multiple surgeries with limited clinical benefit [4]. A possible solution for enhanced diagnostic accuracy and treatment of FRI is offered by bacteria-targeted molecular imaging modalities, in particular fluorescence imaging (FLI) and positron emission tomography combined with computed tomography (PET/CT) [5–8]. Vancomycin, a glycopeptide antibiotic, is a promising candidate for the purpose of bacteria-targeted imaging as it binds to the peptidoglycan of Gram-positive bacteria, which are most frequently associated with FRIs [9].

FLI is thought to be ideal for the visualization of bacterial infections during surgery but, for this purpose, effective bacteria-targeted fluorescent tracers are required [10–12]. Our recent studies suggest that vancomycin-IRDye800CW, a conjugate of the bacteria-specific antibiotic vancomycin and the near infrared (NIR) fluorophore IRDye800CW [13, 14], is suitable for the imaging of FRI [15]. This tracer was shown to be very effective in the visualization of Gram-positive bacteria in in vivo murine infection models and on infected clinical osteosynthesis materials ex vivo [14, 15].

In the clinic, [^18^F]fluorodeoxyglucose ([^18^F]FDG) with PET and labeled white blood cell scintigraphy (WBC) combined with single-photon emission computed tomography (SPECT) are presently the imaging modalities of choice for imaging-based diagnosis of infection prior to surgery. However, these modalities lack sensitivity and are unable to distinguish infected from inflamed tissues ([^18^F]FDG-PET/CT), or are logistically complex and visualize only secondary markers of infection (WBC SPECT/CT) [16–18]. Recent studies have, therefore, addressed the development of bacteria-targeted PET tracers to improve the diagnostic accuracy of infection imaging [8, 19]. Inspired by the positive results of our preclinical studies with the fluorescent vancomycin-IRDye800CW tracer, we recently developed PET tracers based on vancomycin, including [^18^F]PQ-VE1-vancomycin [5, 20, 21]. As expected, [^18^F]PQ-VE1-vancomycin was effective in the visualization of Gram-positive bacterial infection in an in vivo murine myositis infection model and allowed the distinction between bacterial infection and sterile inflammation.

Since it remained unknown how bacteria-targeted tracers for FLI or PET imaging can be implemented in the clinical routine, the aim of our present study was to apply vancomycin-IRDye800CW [13] and [^18^F]PQ-VE1-vancomycin [20] for the visualization of Gram-positive bacterial biofilms on osteosynthesis plates and their surgical debridement in a human post-mortem model of FRI. This set-up was preferred over a preclinical animal model, as it allowed us to closely mimic the clinical debridement workflow. In doing so, we posed the following two research questions: (1) Can [^18^F]PQ-VE1-vancomycin be used in bacteria-targeted PET imaging for the non-invasive detection of bacterial biofilms on osteosynthesis plates before and after surgical debridement? (2) Can fluorescence imaging with vancomycin-IRDye800CW detect and quantify bacterial biofilms on osteosynthesis plates during routine surgical debridement?

**Fig. 1:**
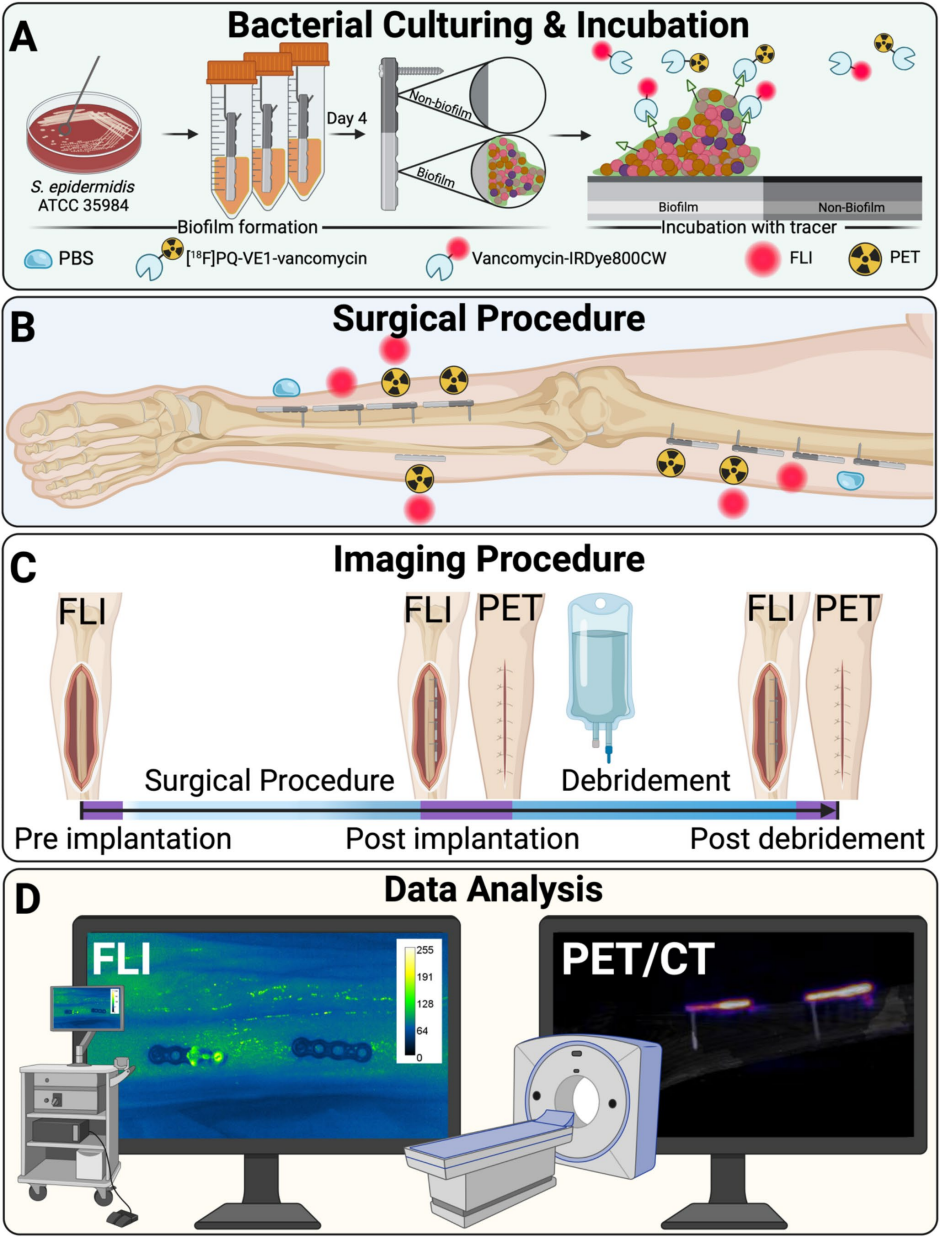
Schematic representation of the procedures for bacteria-targeted vancomycin-based fluorescence-guided surgery and PET imaging of infected osteosynthesis plates in a human post-mortem model. **(A)** Osteosynthesis plates with one half covered with a bacterial biofilm and the other half left uncoated were prepared by dipping the plates in a culture of *S. epidermidis* ATCC 35,984. The osteosynthesis plates were then incubated with the vancomycin-IRDye800CW and/or [^18^F]PQ-VE1-vancomycin tracers and washed to remove any tracer that was not bound. **(B)** The osteosynthesis plates with *S. epidermidis* biofilms were placed on the femur and tibia and secured with a single screw. An osteosynthesis plate extracted from a patient with a confirmed *S. aureus* infection and preincubated with both tracers was secured on the fibula with sutures. **(C)** During the experiment, FLI and PET images were acquired for quantification of the bacterial biofilm. FLI was performed on the open wound, whereas PET imaging was performed after the wound was closed by suturing. **(D)** Both FLI and PET data were collected and analyzed to determine accumulation of signal

**Fig. 2:**
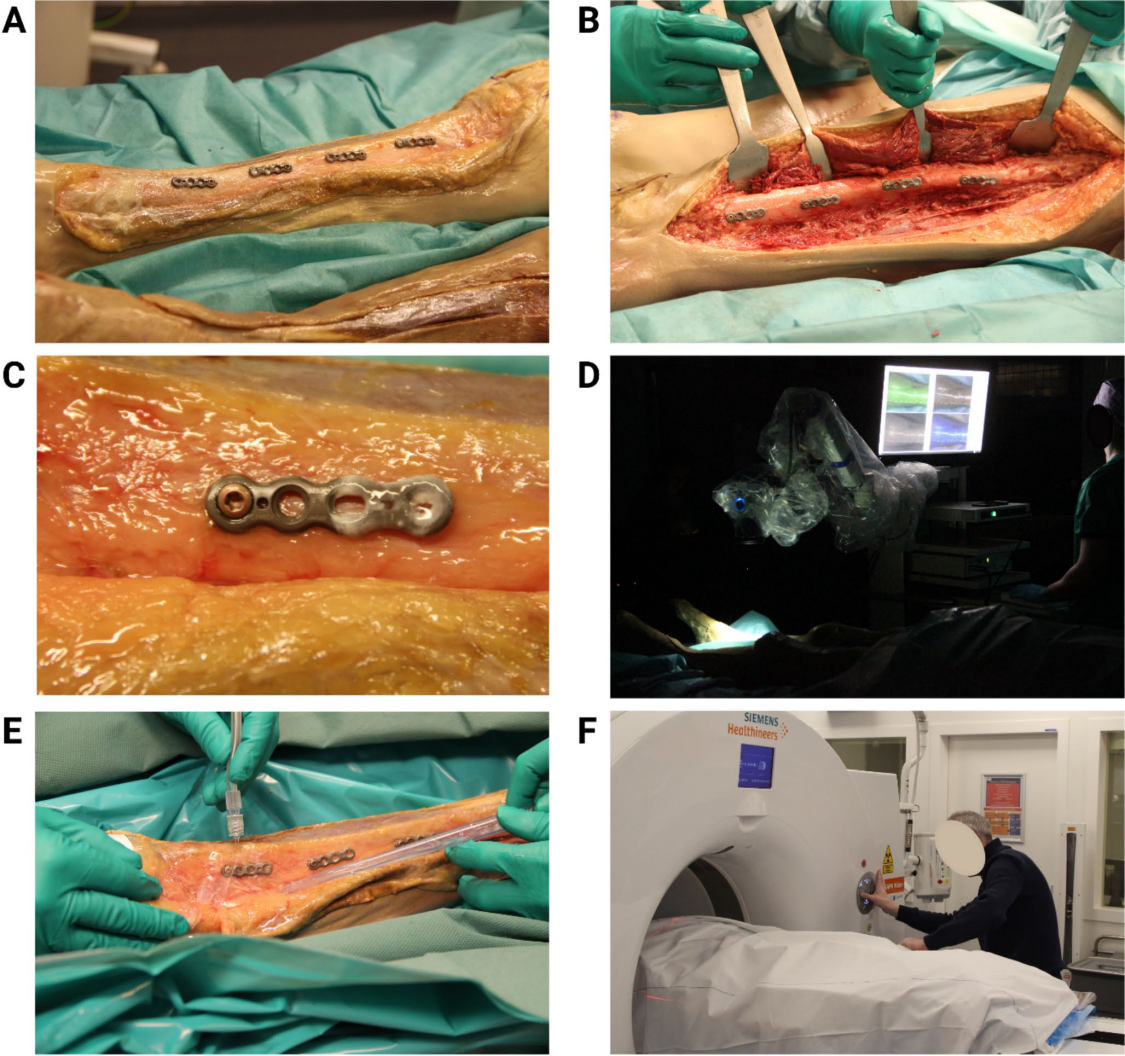
Images illustrating the overall experimental procedure, starting with osteosynthesis plate placement on the tibia **(A)** and the femur **(B)**. Bacterial biofilm was present on half of each implanted device **(C)**. **(D)** shows the Quest fluorescence imaging system with images shown in real-time on the computer display. The bottom images show surgical debridement of the proximal tibia plate, using saline **(E)**, and preparation for the PET scan **(F)**

## Materials and methods

### Study design

A full-body Thiel-embalmed human cadaver specimen from the University Medical Center Groningen (UMCG) Skills Center was used for the present post-mortem infection imaging study [22]. All *post-mortem* experiments were performed *in duplo*, using both legs of the cadaver specimen. The deceased had provided written consent for the post-mortem use of the body for research and education purposes. The post-mortem experiment was conducted at the Wenckebach Skills Center of the UMCG in accordance with the applicable law (“Wet op de Lijkbezorging,” Art 18, lid 1 and 19, BWBR0005009) and UMCG guidelines.

We performed surgery on the tibia and femur, applying a total of 16 VariAx 2 titanium 4-hole compression plates (Stryker, Kalamazoo, USA) for osteosynthesis, with four plates each on the left femur, left tibia, right femur, and right tibia. Each plate was partially covered with biofilm and incubated with either phosphate-buffered saline (PBS), vancomycin-IRDye800CW, [^18^F]PQ-VE1-vancomycin, or both vancomycin-IRDye800CW and [^18^F]PQ-VE1-vancomycin. A surgical debridement was performed, following the clinical recommendations for treating fracture-related infections as described by Metsemakers et al. [23]. At various time points, FLI and PET/CT scans were performed to detect and quantify bacterial infection on the implanted osteosynthesis plates before and after surgical debridement. A schematic representation of the procedures is depicted in Fig. 1. PET data was quantified in Bq/mL, and FLI in mean fluorescence intensity (MFI, arbitrary units (AUs)), per implant side. Subsequently, ratios were calculated between the biofilm-covered side and the sterile side of the osteosynthesis plate.

### Bacterial culturing and biofilm formation on osteosynthesis plates

The Gram-positive bacterium *Staphylococcus epidermidis* (*S. epidermidis)* ATCC 35,984 was used in this study because of its high capacity to form biofilm [24]. Bacteria were cultured in Tryptic Soy Broth (TSB) supplemented with 2.5% glucose and 2.5% NaCl (TSB^+^). A day culture was prepared by inoculating 10 mL supplemented TSB^+^ at an OD_600_ of 0.05. After ± 1.5 h of incubation at 37 °C and 250 RPM, the now exponentially growing bacterial culture was used for the inoculation of the osteosynthesis plates at an OD_600_ of 0.05. Bacterial biofilms were grown on the above-mentioned titanium osteosynthesis plates, as shown in Fig. 1A. To this end, these plates were suspended from a stainless steel Kirschner wire (K-wire) at a height where half of each plate was submerged in TSB^+^ culture medium that had been inoculated with bacteria. The remaining half was not in contact with the medium to serve as a non-biofilm control. Each day, the osteosynthesis plates were moved to a container with fresh TSB^+^ medium (without bacteria) until sufficient biofilm had accumulated on their surface, which was the case on day 4. Macroscopically, the submerged side of the plates developed an off-white, fuzzy biofilm layer, indicating the presence of adherent bacteria. No biofilm was detectable on the plates’ side that had not been in contact with the bacteria-containing medium. For the in vitro experiments, larger osteosynthesis plates (SPS; Stryker Plating System) one-third tubular plate, 8 holes, l = 103 mm, and rectangular blanks were prepared by culturing in a rectangular petri dish, using sufficient culture medium (inoculated with *S. epidermidis*, or maintained in sterile fashion). The plates and blanks were placed on 0.8 mm stainless steel (SST) Kirschner wires, to minimize direct contact with the bottom of the petri dish. Each day, the plates were moved to a petri dish with fresh TSB^+^ medium until sufficient biofilm had accumulated on their surface, which was the case on day 4.

### Production of PET tracer and NIR fluorescent imaging tracer

[^18^F]PQ-VE1-vancomycin was prepared as previously described [5, 20]. Vancomycin-IRDye800CW was prepared by Symeres (Groningen, The Netherlands) as previously described [13].

### Incubation of the osteosynthesis plates with fluorescent and/or PET tracers

Once biofilms had developed (on day 4), the osteosynthesis plates were removed from the culture vessel and washed by suspending the osteosynthesis plate in sterile PBS with 0.1% bovine serum albumin (BSA) to remove planktonic bacteria and culture medium. BSA was added to the PBS to mimic the wound environment, and to minimize non-specific accumulation of tracer. After washing, the osteosynthesis plates were incubated with either [^18^F]PQ-VE1-vancomycin, vancomycin-IRDye800CW, a combination of both tracers, or PBS, for a total of 20 min at room temperature. Each osteosynthesis plate was incubated with 0.3 MBq · mL^− 1^ of [^18^F]PQ-VE1-vancomycin, and/or 0.14 µM of vancomycin-IRDye800CW in 10 mL PBS, or PBS alone (Fig. 1A). For the in vitro experiment, each specimen was incubated with 1 MBq of [^18^F]PQ-VE1-vancomycin in sufficient PBS to fully submerge the materials. After incubation, the osteosynthesis plates were washed twice in PBS with 0.1% BSA to remove any unbound tracer. The washing procedure consisted of gentle submersion in the washing container, whilst trying to minimize disruption of the bacterial biofilms. Thereafter, the plates were ready for use in the surgical procedure, or in vitro experiments.

### Surgical procedure and data collection

The human cadaver leg was disinfected and draped. An anteromedial incision was made over the tibia, and the lateral side of the femur, exposing both bones. Four biofilm-covered plates (Stryker, Variax 2-foot broad straight plate, 4-holes, l = 35.5 mm x w = 8.5 mm) were placed on the tibia and four on the femur of each leg. The plates were fixed to the bone using a single VariAx 2 T10 bone screw (3.5 mm x l = 26 mm) on the non-biofilm side of the plates (Figs. 1B and 2). A separate incision was made over the proximal fibula and the bone was exposed. To investigate whether our FLI and PET tracers also detect clinical biofilms in the *post-mortem* model, a plate extracted from a patient who had undergone revision surgery for FRI was placed on the fibula (METc 2017/526). The patient suffered from a confirmed *Staphylococcus aureus* (*S. aureus*) infection. Subsequently, FLI and PET/CT were performed at fixed time-points during the procedure to determine the potential to detect bacterial biofilms with our [^18^F]PQ-VE1-vancomycin PET tracer, and to quantify the efficacy of routine implant debridement in the removal of bacterial biofilms using our fluorescent vancomycin-IRDye800CW tracer (Figs. 1C and 2). FLI was performed at different instances during the first ‘diagnostic stage’ of the procedure: prior to and after osteosynthesis plate placement and after closure of the surgical site using Ethilon 3 − 0 (Ethicon, Somerville, USA). Measurements were performed using a Quest Spectrum fluorescence imaging system (Quest Medical Imaging B.V., Middenmeer, The Netherlands), held at a standardized distance of 30 cm from the surgical field. The camera was set to a gain of 22.5 dB and exposure times of 400 ms and 1000 ms. The detection limit of the Quest Spectrum, used for detection of the NIR fluorophore IRDye800CW was previously determined to be approximately 10 nM under ideal conditions [25, 26]. Subsequently, low dose CT and ultra-high sensitivity (UHS) PET was performed with the osteosynthesis plates in situ and closed wounds. Measurements were performed on a Biograph Vision Quadra system (Siemens Healthineers, Knoxville, USA). The first PET/CT scan was performed for a duration of 30 min. At the second ‘debridement stage’ of the procedure, the wound was reopened. Surgical debridement with 3 L of 0.9% saline was performed mimicking the standard of care clinical debridement (Fig. 2). Flowrate of the saline solution was regulated by gravity, and a sterile gauze was used during the debridement to dislodge bacterial biomass from the osteosynthesis plates. Afterwards, FLI was performed to quantify the efficacy of routine debridement and to detect any residual bacterial biofilm. Subsequently, the wounds were closed. The second PET/CT scan was performed for a duration of 60 min, marking the end of the procedure. In total, the experiment was performed over a time period of approximately 8 h from the start of incubation of the implants with tracer until the last measurement.

### Data analysis

**Fig. 3:**
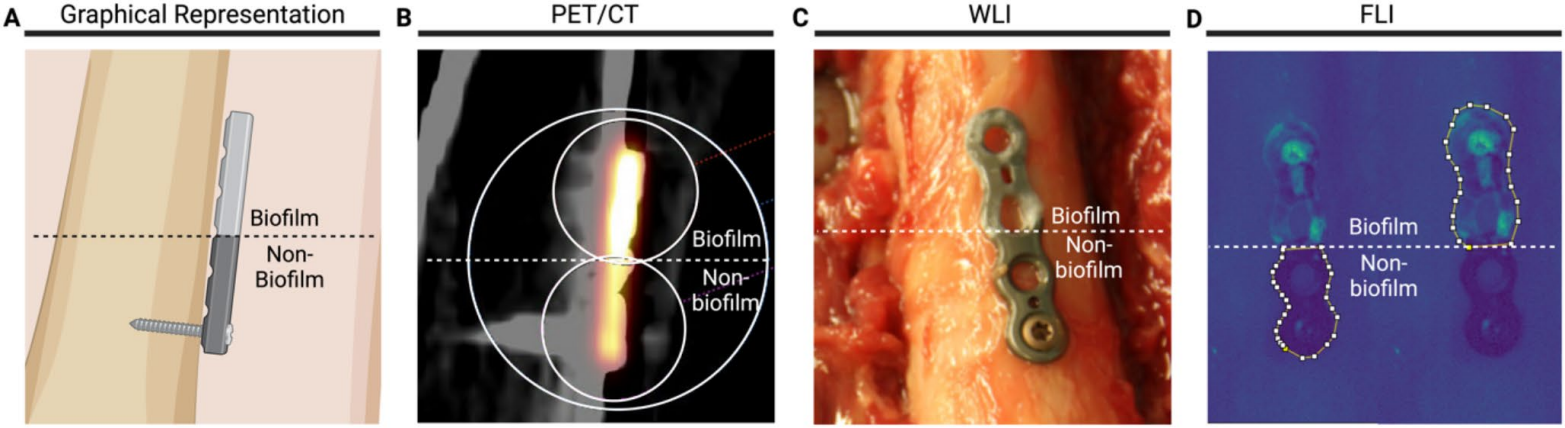
PET imaging and FLI of an osteosynthesis plate mounted on a human femur. **(A)**: Graphical representation of femur and osteosynthesis plate. The non-biofilm side of the implant is oriented towards the bottom of the image, and the biofilm-covered side is oriented towards the top of the image. **(B)**: PET/CT image of the osteosynthesis plate. The screw can be seen in the lower half of the image, protruding from right to left into the femoral cortex. VOIs are shown for the biofilm-covered side, non-biofilm side, and entire osteosynthesis plate. The biofilm-covered side (top) visually shows the highest tracer activity. **(C)**: White light image (WLI) of the osteosynthesis plate in situ with biofilm present on the top half of the implant. **(D)**: FLI of the osteosynthesis plate. The yellow line indicates the regions of ROIs for the non-biofilm side and biofilm side, respectively

PET images collected were analyzed using Syngo.via software suite (version VB60 HF05, Siemens Healthineers, Erlangen, Germany). Volumes of interest (VOIs) were drawn (two with a diameter of 2.4 cm, encompassing each half of the osteosynthesis plate, and a third VOI was drawn with a diameter of 5.0 cm, encompassing the entire osteosynthesis plate) and exported as.csv files for further analysis. Fluorescence images collected with the Quest Spectrum fluorescence imaging device were analyzed using ImageJ (version 2.54d). Regions of interest (ROIs) were drawn and exported as.csv files (Fig. 3). Specifically, the osteosynthesis plates’ outline was traced using a polygon tool, making sure to differentiate the non-biofilm side from the biofilm-coated side. After debridement, this distinction was no longer visible, and the osteosynthesis plates’ midline was used as border between the non-biofilm side and the biofilm-coated side. The resulting files were imported into R Studio (version 2024.04.2 + 764, Posit PBC, Boston, USA). A non-parametric Mann-Whitney *U* test was performed to determine statistical significance, comparing accumulated signal (PET or FLI) pre- and post-debridement. Ratios were calculated by dividing target signal by non-target signal. A *p*-value of < 0.05 was considered to indicate statistical significance.

## Results

### Noninvasive detection and quantification of bacterial biofilms on osteosynthesis plates using [^18^F]PQ-VE1-vancomycin

**Fig. 4:**
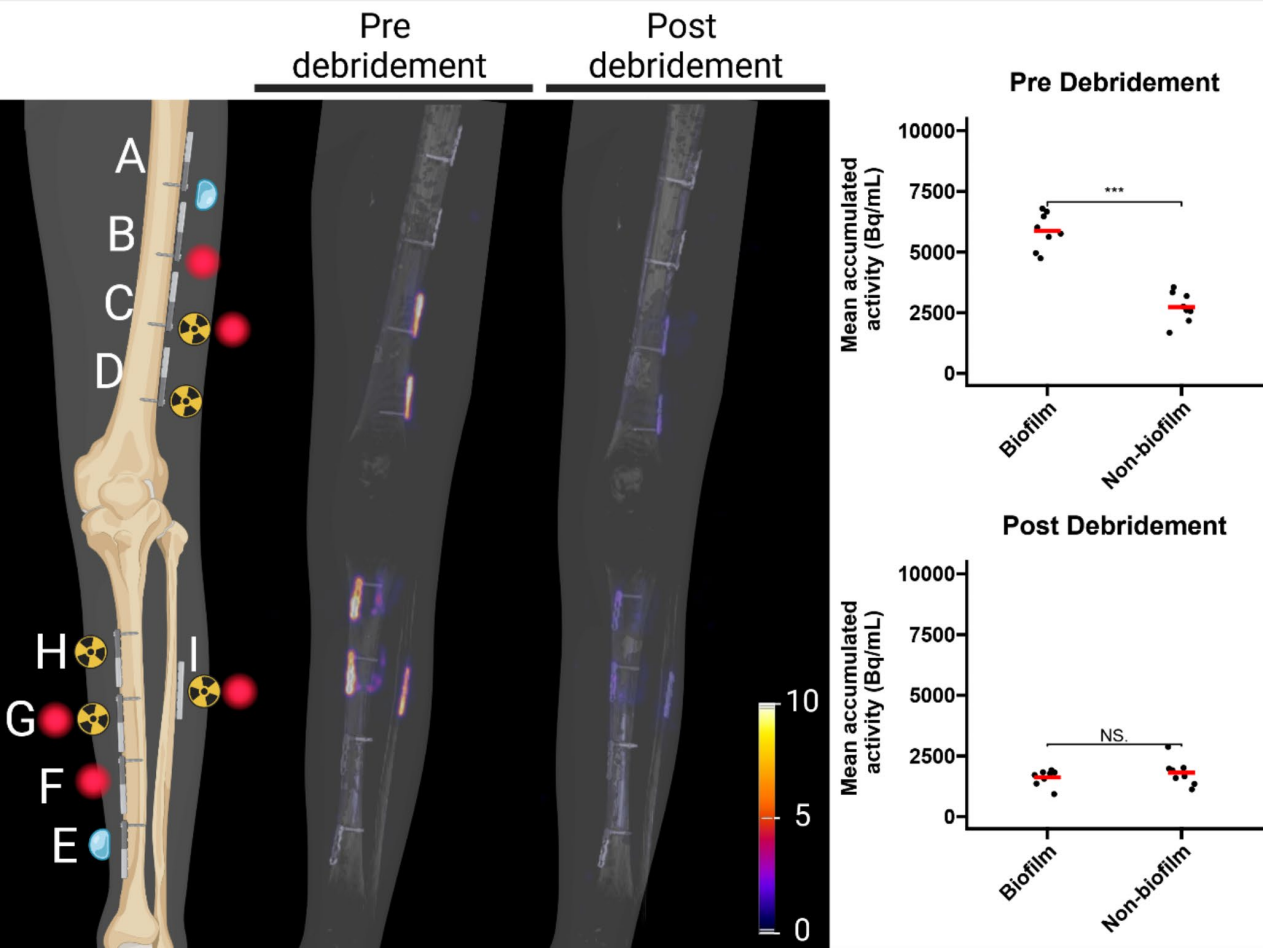
[^18^F]PQ-VE1-vancomycin can be used to detect the presence of bacterial biofilms using non-invasive PET/CT. **Left**: Schematic representation of the placement of biofilm-bearing osteosynthesis plates and their pre-incubation with vancomycin-based PET or FLI tracers, and PET/CT images for pre- and post-debridement, showing that the PET signal observed before debridement on the implanted plates C, D, G, and H, is caused by accumulation of tracer on the *S. epidermidis* biofilms. After removal of the biofilms through a routine surgical debridement, virtually no residual radioactivity was observed. The same is true for the osteosynthesis plate I, which was extracted from a patient with a confirmed *S. aureus* infection that was mounted on the fibula. The color legend represents kBq/mL. **Right**: Quantification of PET signal on the implanted plates C, D, G, and H expressed as mean accumulated activity (Bq/mL). The red bar indicates the mean value. Before surgical debridement, 2.2 (± 0.6) fold more tracer was observed to accumulate on the biofilm-covered side of the plates than on the non-biofilm side. After surgical debridement, this ratio was reduced to 0.9 ± 0.3. Comparing the biofilm-covered side before and after debridement resulted in a ratio of 3.6 ± 0.9. ***, *p* < 0.001. NS, not significant

To explore the possibilities of bacteria-targeted imaging with PET/CT, experiments were performed using the PET tracer [^18^F]PQ-VE1-vancomycin [20]. Osteosynthesis titanium plates that were half covered with a biofilm of the Gram-positive bacterium *S*. *epidermidis* were incubated with [^18^F]PQ-VE1-vancomycin and the retention of this tracer was measured at two timepoints in a clinical PET/CT scanner, once before surgical debridement and once after debridement (Figs. 1 and 2). For quantification of the signal, three VOIs were drawn around the image of each osteosynthesis plate (Fig. 3). After decay correction, the accumulated signal was calculated as mean activity (Bq/mL). Figure 4 shows an overview of the gathered CT and PET images, as well as the mean activity for both the non-biofilm and biofilm-coated sites of the plates. These images show that using [^18^F]PQ-VE1-vancomycin it was possible to effectively localize the bacterial biofilm on the osteosynthesis plates before surgical debridement, resulting in 2.2 (± 0.6) fold more tracer accumulation on the biofilm-covered side compared to the non-biofilm side. After surgical debridement with saline (3 L), as in the standard clinical practice, [^18^F]PQ-VE1-vancomycin signals were no longer detected, suggesting that most of the biofilm had been removed effectively (Fig. 4).

**Fig. 5:**
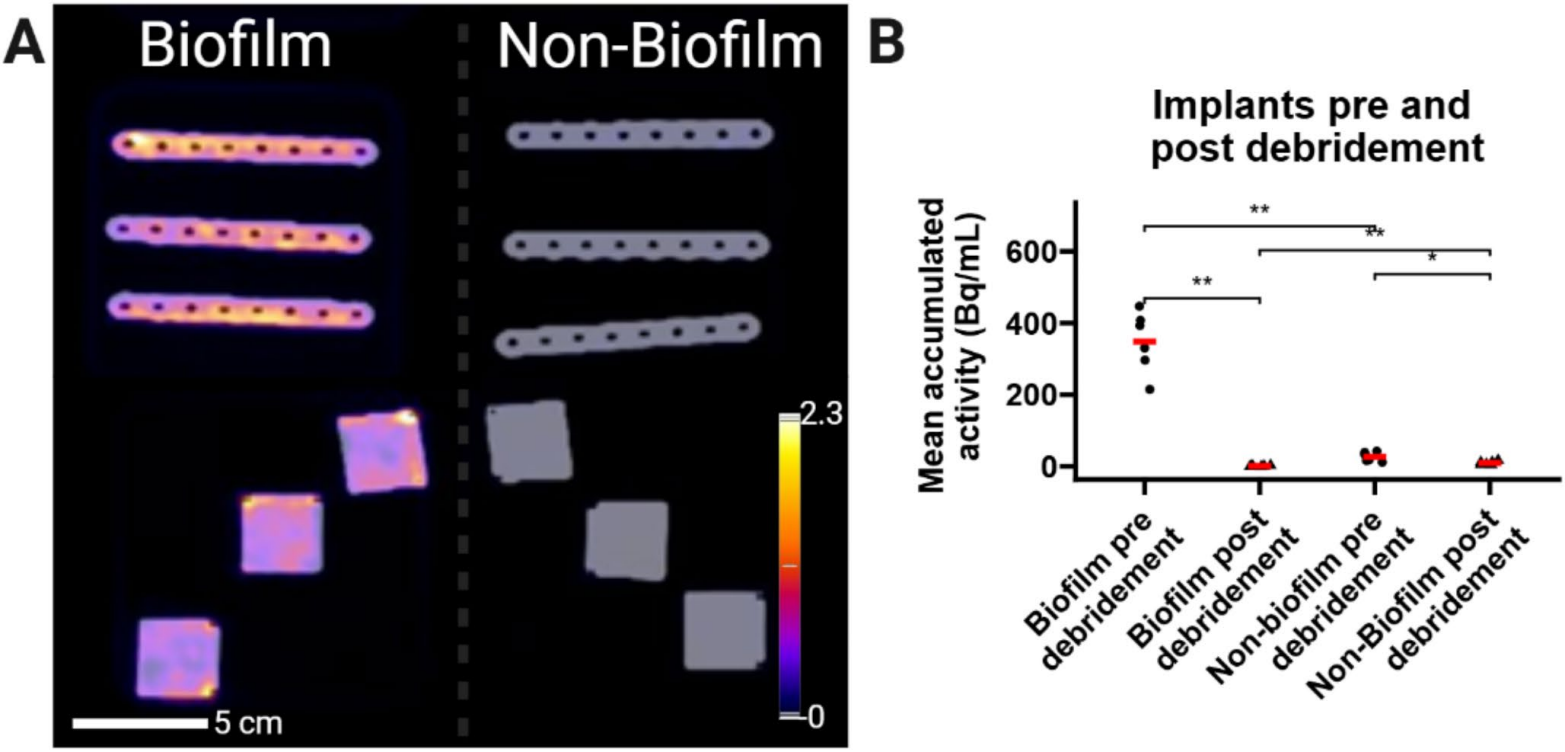
**(A)** PET/CT images showing the osteosynthesis plates used for the additional experiments. The images show accumulation of tracer on the plates that carry a bacterial biofilm. Values are expressed as kBq/mL. **(B)** Quantification of the in vitro data (mean accumulated activity in Bq/ml) validates the findings in the post-mortem experiment, showing that bacterial biofilms are effectively detected using the PET tracer, and that the effect of debridement can be visualized using this PET tracer in the presently applied human post-mortem implant infection model. *, *p* < 0.05. **, *p* < 0.01

To verify the binding of [^18^F]PQ-VE1-vancomycin to biofilm-coated osteosynthesis plates, the experiment was repeated using osteosynthesis plates that were fully covered in biofilm, or were kept entirely sterile. Larger osteosynthesis plates and rectangular material blanks were selected for these additional experiments. Consistent with the results described above, increased activity was detected on the biofilm-coated osteosynthesis plates (Fig. 5).

### Intraoperative visualization of the extent of bacterial biofilm removal after surgical debridement using a vancomycin-based fluorescent tracer

**Fig. 6:**
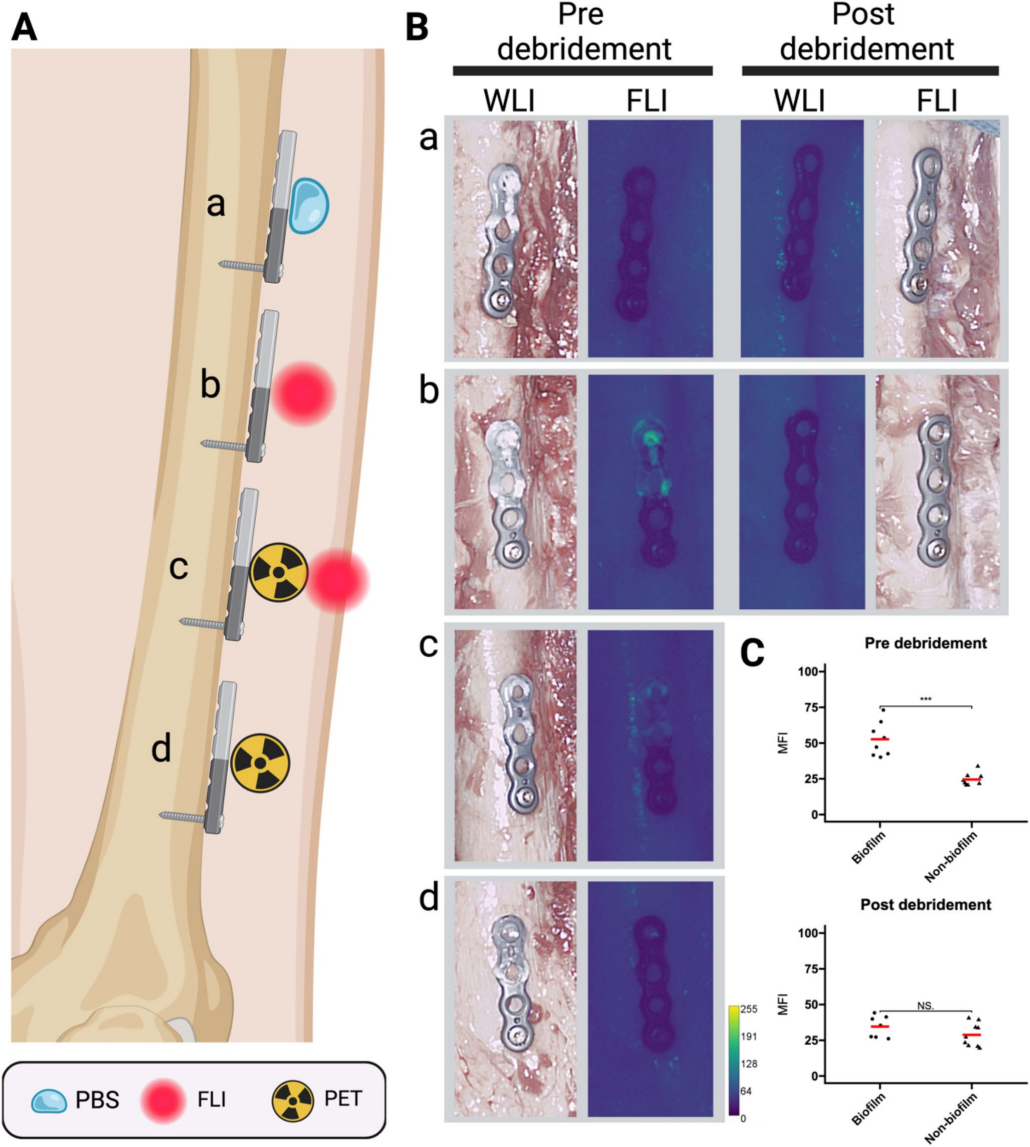
**(A)** Representation of biofilm-coated osteosynthesis plates, incubated with or without vancomycin-IRDye800CW. **(B)** Accumulation of fluorescent tracer is visible on the biofilm-covered side (proximal side) of the osteosynthesis plates incubated with vancomycin-IRDye800CW (plate b), or a combination of vancomycin-IRDye800CW and [^18^F]PQ-VE1-vancomcyin (plate c), but not on plates incubated with PBS (plate a, negative control) or only [^18^F]PQ-VE1-vancomycin (plate d). Moreover, slight light reflections on the bone can be observed adjacent to the plates (plate a and c). After debridement, the biofilms were no longer visible on the exposed side of the implant (WLI and FLI, post debridement). The color bar represents fluorescence intensity (arbitrary units). **(C)** Mean fluorescence intensity (MFI) was compared before and after the debridement procedure. Routine debridement of infected lesions effectively removed bacterial biofilms from the exposed surfaces. The MFI was determined for osteosynthesis plates incubated with vancomycin-IRDye800CW. Before debridement, the biofilm-covered side demonstrated 2.1 (± 0.6) fold more fluorescence signal compared to the non-biofilm-covered side. After debridement, this ratio was reduced to 1.2 ± 0.4. Comparing the biofilm-covered side before and after debridement, a ratio of 1.5 ± 0.5 was observed. ***, *p* < 0.001. NS, not significant

Osteosynthesis plates incubated with vancomycin-IRDye800CW or a combination of vancomycin-IRDye800CW and [^18^F]PQ-VE1-vancomcyin showed a fluorescence signal on the biofilm-covered side, confirming that biofilms can effectively be identified perioperatively using FLI (Fig. 6). The biofilm-covered side emitted a fluorescence signal that was 2.1 (± 0.6) fold higher compared to the non-biofilm side. The implant sides not covered with biofilm showed no fluorescence signal, regardless of the incubation medium (PBS, fluorescent tracer, nuclear tracer, or both). This implies that vancomycin-IRDye800CW shows specific binding to the bacterial biofilm, enabling effective differentiation between infected and non-infected areas.

To test whether vancomycin-IRDye800CW can be used to monitor the success of surgical debridement with respect to the removal of bacterial biofilm from an osteosynthesis plate, for each surgical site (i.e. femur and tibia) the respective plates were debrided using 3 L saline solution. FLI was performed after debridement, and the total residual tracer signal was quantified from a ROI drawn around the osteosynthesis plate outline. As shown in Fig. 6C, most of the biofilm was effectively removed from the visible sides of the osteosynthesis materials, as there was no longer a significant fluorescence signal detectable after debridement.

**Fig. 7:**
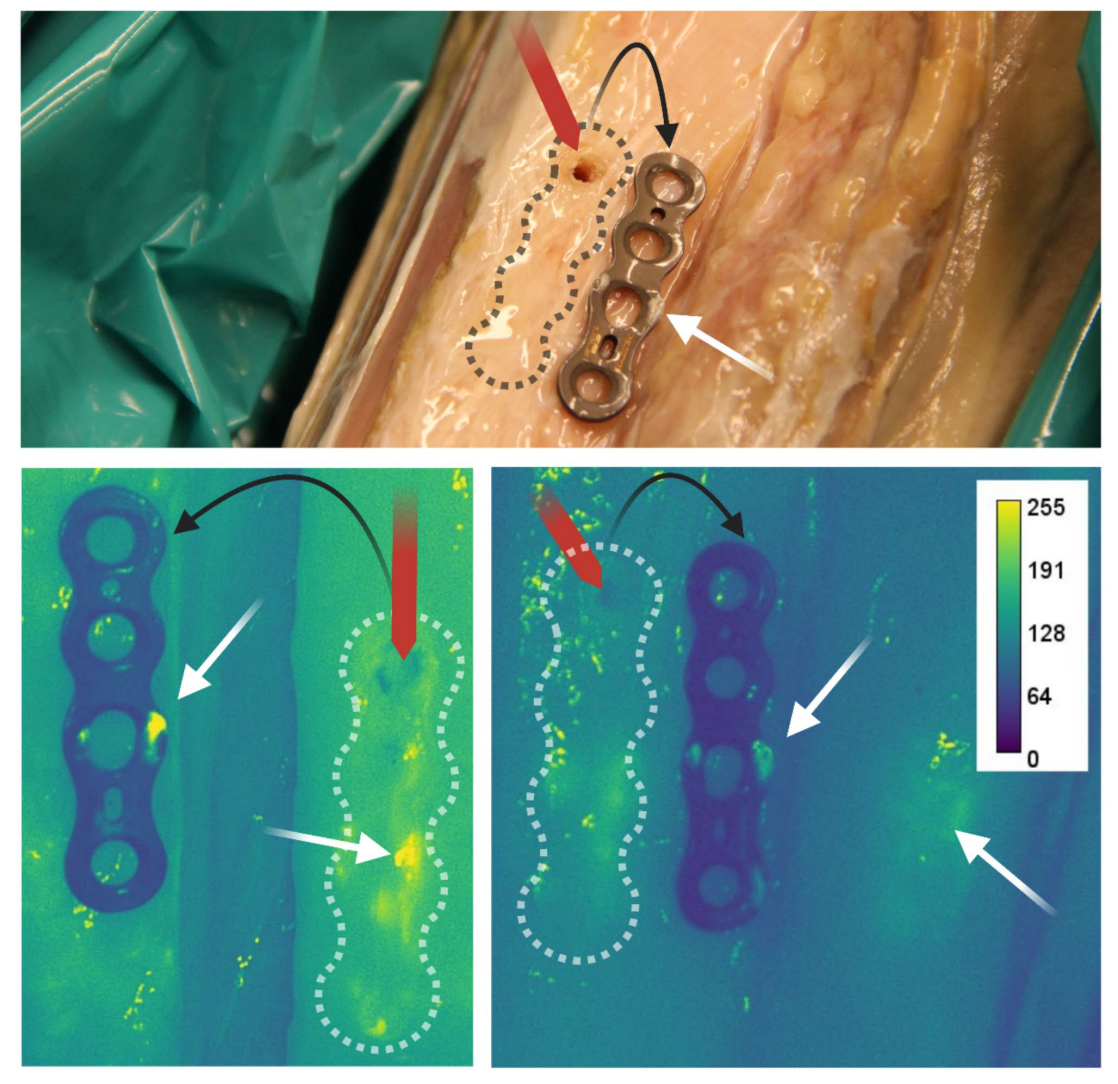
Detection of residual *S. epidermidis* biofilm at the osteosynthesis plate-bone interface on plates incubated with vancomycin-IRDye800CW. An *S. epidermidis* biofilm was grown in vitro, mounted on the tibia, and debrided as described in Figs. 1 and 2. After debridement, the undersurfaces of the osteosynthesis plates were inspected by removing the screw and exposing the underside of the osteosynthesis plate (**top**). The outline of the original osteosynthesis plate location and screw hole is indicated with the dotted line and red arrow, respectively. Biofilm remnants on both the osteosynthesis plate and osteosynthesis plate-bone interface were clearly visible (white arrows) using fluorescence imaging (**bottom**). These findings suggest the presence of biofilm remnants at the interface of the osteosynthesis plate and the bone after debridement, despite the removal of all visible biofilm traces from the exposed side of the osteosynthesis plate. The color bar represents fluorescence intensity (arbitrary units)

Since the biofilm was grown both on the exposed surface and the opposite surface facing the bone, osteosynthesis plates incubated with vancomycin-IRDye800CW were removed following the debridement and inverted to expose the osteosynthesis plates’ undersurface (Fig. 7). Interestingly, the presence of biofilm was evident on both the osteosynthesis plates’ undersurface, as well as the bone interface to which the osteosynthesis plate had been affixed. This implies that biofilms on the osteosynthesis plates’ undersurface are difficult to remove by routine debridement, which is in line with the fact that fluorescence signals cannot penetrate metal, hindering in situ imaging of the undersurface of the osteosynthesis materials.

**Fig. 8:**
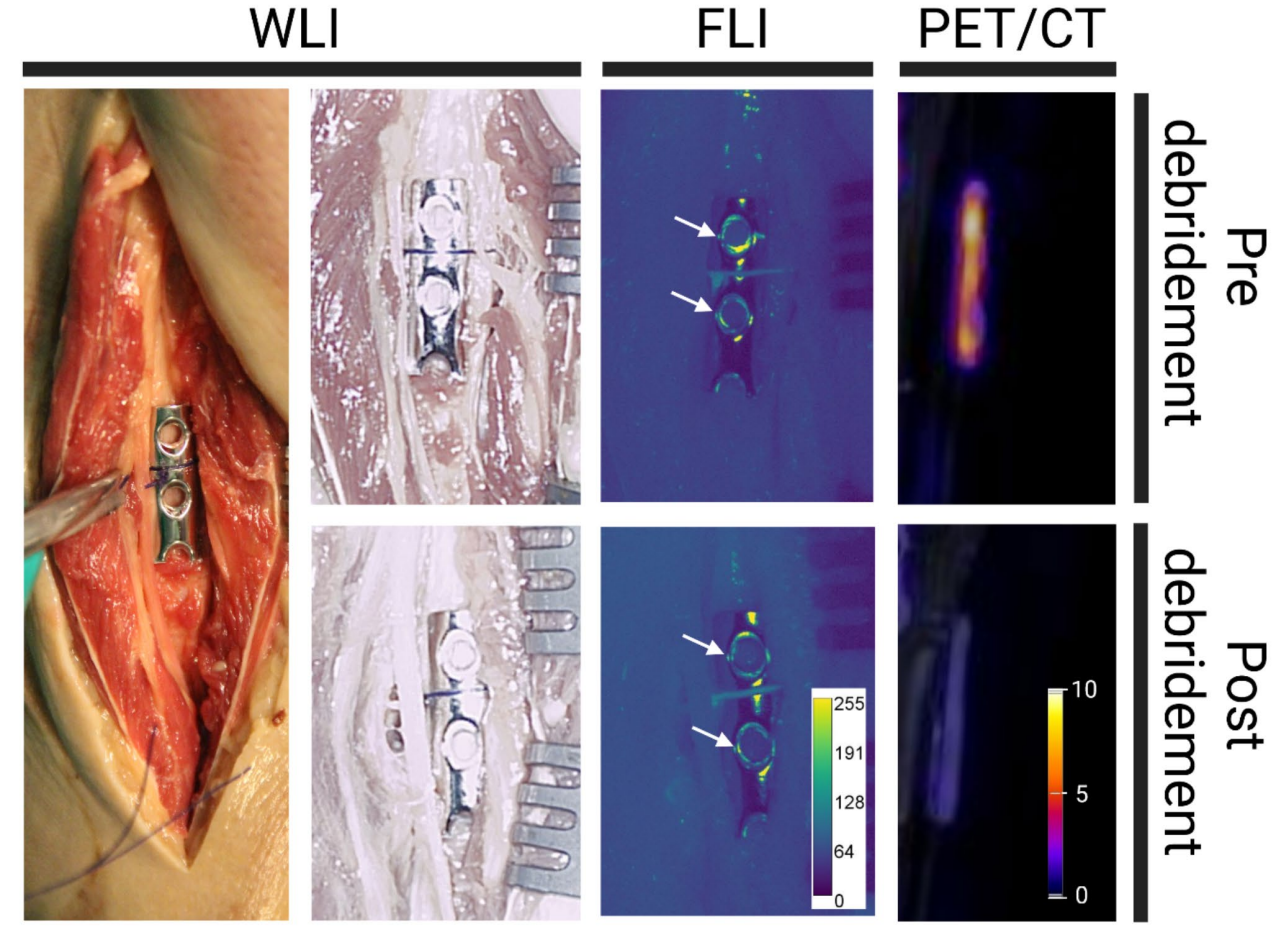
An stainless steel osteosynthesis plate was extracted from a patient with a confirmed *S. aureus* fracture-related infection and placed on the human cadaver fibula. Using the same approach as for imaging of the osteosynthesis plates on the femur and tibia, the plates were imaged with vancomycin-IRDye800CW and [^18^F]PQ-VE1-vancomcyin using FLI and PET/CT. Due to the surface finish of this plate, some reflections are visible around the midline of the plate in the case of FLI. Specific tracer uptake is visible around the screw holes, pre debridement (marked by arrows). Post debridement, the reflections remain visible by FLI, but the signal around the screw holes is reduced (see arrows). A similar pattern is clearly visible for the PET/CT images, where increased radioactivity is detected pre debridement, but is largely removed through the debridement. In the PET/CT image, the plate is viewed from the side

The experiments described so far were performed with in vitro generated biofilms. To replicate the imaging of infections that mimic actual clinical FRIs, two stainless steel osteosynthesis plates extracted from a patient with a confirmed *S. aureus* infection were included in our post-mortem study. These plates were processed the same way as the osteosynthesis plates with in vitro grown *S. epidermidis* biofilms and fixated to the fibula using sutures (Fig. 1B). The results presented in Figs. 4 and 8 show the detection of the bacterial biofilm by PET/CT and FLI, respectively. As evidenced by FLI, the biofilm resided predominantly around the plates’ screw holes (Fig. 8). This finding is corroborated by PET/CT, showing tracer accumulation in the same areas. Post debridement, the majority of biofilm was effectively removed, as indicated by both PET/CT and FLI (Figs. 4 and 8).

## Discussion

In the present study, we demonstrate that vancomycin-based PET and fluorescent tracers can be used in combination for non-invasive and real-time imaging of Gram-positive bacterial infections as encountered in FRIs. By using a human post-mortem infection model, we show that non-invasive PET imaging effectively distinguishes infected osteosynthesis plates from plates without bacterial biofilm. Furthermore, we show that intraoperative fluorescence imaging of infected osteosynthesis plates can guide real-time surgical debridement.

We consider vancomycin as a promising molecule for the development of bacteria-targeted tracers to detect FRI and other infections of implanted biomedical devices, because this antibiotic effectively targets Gram-positive bacteria, which are the most common pathogens involved in biofilm-associated implant infections [9]. At least 80% of fracture-related infections are caused by Gram-positive pathogens [27]. Accordingly, we showed previously that the tracers applied in our present study, vancomycin-IRDye800CW and [^18^F]PQ-VE1-vancomycin, have high affinity for Gram-positive bacteria, including *S. aureus*, *S. epidermidis* and even vancomycin resistant *Enterococcus faecium* [10, 14, 15, 20, 21]. Our present study now pioneers the capabilities of vancomycin-based PET imaging to detect implant infections in the human body by introducing in vitro and in vivo generated bacterial biofilms in a post-mortem body and applying a clinical workflow to precisely localize the implant-borne biofilms. Our present workflow thus closely mimics the clinical setting. Importantly, the results show that with the aid of [^18^F]PQ-VE1-vancomycin it is now possible to non-invasively differentiate biofilm-bearing sites of an implant from biofilm-free sites.

To complement the sensitive whole-body imaging qualities of PET/CT by a high-resolution real-time intraoperative imaging platform, vancomycin-IRDye800CW was used to perform FLI in our human post-mortem implant infection model. Surgeons often find it challenging to visually differentiate non-infected tissue from infected tissue. The preclinical introduction of vancomycin-IRDye800CW marks a significant advancement for detecting Gram-positive bacterial infections in fluorescence-guided surgery [14]. This fluorescent tracer was previously used in various in vitro and in vivo infection models, showing that imaging of Gram-positive bacteria is feasible [10, 14, 15, 21]. Our present study expands the preclinical work on vancomycin-based imaging by incorporating a clinical debridement procedure to simulate the eradication of bacterial biofilms from implanted osteosynthesis plates. Our findings demonstrate that real-time fluorescence-guided surgery can identify biofilms on osteosynthesis plates, and quantify the difference in the bacterial load before and after surgical debridement. Additionally, we show that the use of fluorescent tracers may fail to identify bacterial biofilms located at the bone-facing parts of the osteosynthesis materials when the materials are left in situ, because such tracers cannot be imaged through metal. On the other hand, when the materials are removed, residual biofilm is demonstrated in real-time, pointing out the difficulty to remove bacterial biofilms through surgical debridement. Together, our findings represent a significant advance in the translation of vancomycin-IRDye800CW from preclinical evaluation to clinical application, especially with respect to image-guided tissue sampling and surgical debridement of bacterial biofilms.

It should be noted that our post-mortem human body infection model poses some inherent limitations due to lack of biological functions and metabolic activity. Specifically, it does not simulate blood flow or immune responses around the infected osteosynthesis plates. Due to its lack of blood flow, tracer administration cannot be performed through intravenous injection as is envisaged for the real-life clinical setting. Future research will need to determine whether our vancomycin-based tracers are able to reach the target within the appropriate time frame for the tracer (i.e. bind bacteria in the biofilm), and whether non-specific tracer accumulation in affected tissue is cleared rapidly enough for effective detection of bacterial biofilms in patients. However, if this prerequisite is met, we consider it likely that vancomycin-based fluorescent and PET tracers can be applied for in situ detection of bacterial biofilms because, in our post-mortem setup, we achieved proof-of-principle for the detection of bacterial biofilms on osteosynthesis plates extracted from a patient with a confirmed *S. aureus* infection. This implies that the bacterial load in case of this fracture-related infection was high enough to allow in situ detection by PET/CT, and also by FLI during image-guided surgical debridement. The tracers described here, or improved iterations thereof, have the potential to be applied in other surgical fields, e.g. vascular surgery, abdominal surgery, or surgical oncology, where infections can have severe consequences. Future research should therefore focus on the application and feasibility of these novel vancomycin-based imaging agents and modalities in the clinical setting, in order to evaluate their effectiveness in the reliable detection of residual biofilm upon surgical implant debridement.

## Conclusion

In this study, we present an innovative combination of bacteria-targeted PET/CT imaging and fluorescence imaging. Our new vancomycin-based PET tracer [^18^F]PQ-VE1-vancomycin paves the way for detection and quantification of fracture-related bacterial infections, whereas fluorescence imaging provides intraoperative feedback on the presence of bacterial biofilms, before and after surgical debridement. In the future, treatment of bacterial infections may be achieved with higher quality and at lower costs through advancements in precise, bacteria-targeted diagnostics and surgical techniques. Further clinical trials are necessary to validate these results.

## Data Availability

The datasets used and/or analyzed during the current study are available from the corresponding author on reasonable request.
